# First person – Judith S. Cruz Ortega

**DOI:** 10.1242/bio.043778

**Published:** 2019-04-15

## Abstract

First Person is a series of interviews with the first authors of a selection of papers published in Biology Open, helping early-career researchers promote themselves alongside their papers. Judith S. Cruz Ortega is first author on ‘Actin cytoskeleton remodeling defines a distinct cellular function for adhesion G protein-coupled receptors ADGRL/latrophilins 1, 2 and 3’, published in BiO. Judith is a PhD student in the lab of Antony Jr Boucard, at Centro de Investigación y de Estudios Avanzados del Instituto Politécnico Nacional, Mexico, investigating the molecular basis of brain cellular communication.


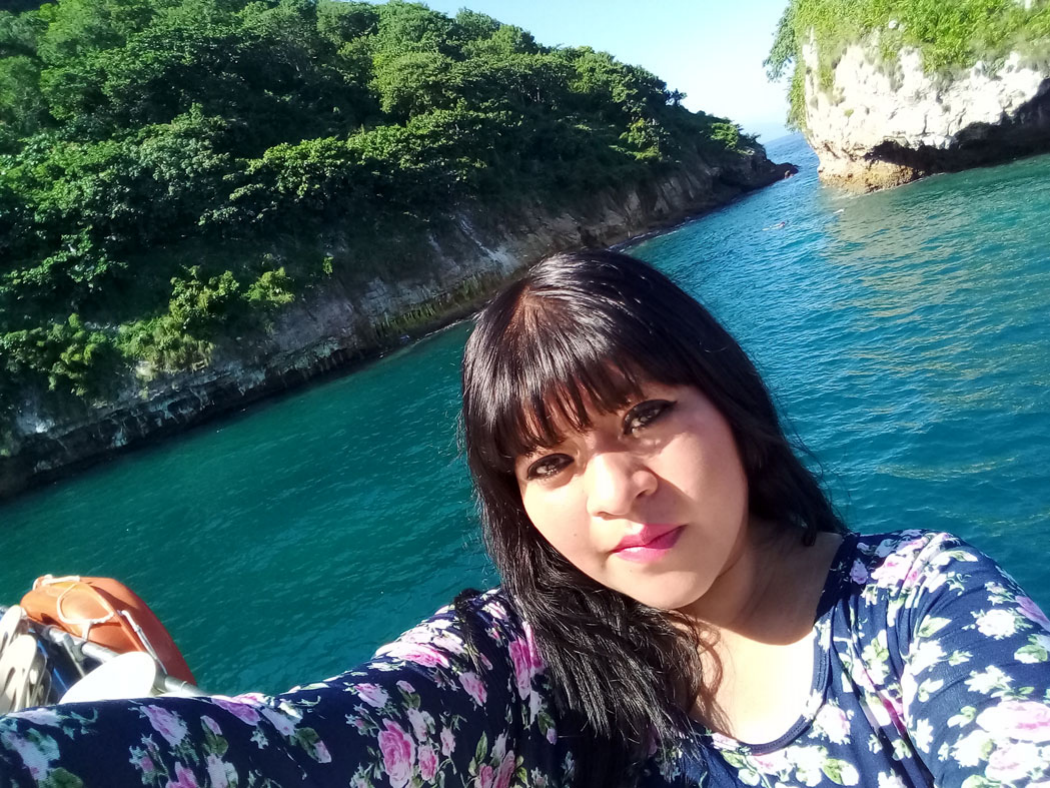


**Judith S. Cruz Ortega**

**What is your scientific background and the general focus of your lab?**

My heart is in science. From very early in my life I entered the world of biological sciences. During my undergraduate studies as an industrial chemist and pharmacobiologist, I participated in research in the field of clinical biochemistry and neuroscience, studying biochemistry and physiological processes such as oxidative stress during stroke, and metabolic dysfunctions such as diabetes. This scientific trajectory allowed me to graduate with honors from undergraduate levels for which I was awarded a prize of national recognition in my country. My experience working with patients in the clinic improved my social skills to the point where I was able to invest myself in public speaking experiences such as participating in radio interviews aimed at making science approachable to a wider audience – this audience being the people of Puebla, my native city in Mexico. My laboratory's studies focus on investigating the molecular basis of synapse formation in a physiological and pathophysiological setting. Among the different ailments my lab intends to contribute valuable knowledge to are the widely known attention deficit hyperactivity disorder, autism spectrum disorder and bipolar disorder. For this, we incorporate various techniques such as microscopy, molecular biology, cell biology and biochemistry to analyze patient samples from the clinic, animal models and cellular systems, to name a few.

**How would you explain the main findings of your paper to non-scientific family and friends?**

The brain is an organ made up of millions of neurons that interact with each other to carry out complex functions in our body, such as the movement of a muscle, breathing or the generation of a thought. Imagine that each neuron contacts one another by extending their arms and hands in order to touch and lock in each other's fingers. Well, these fingers have to be strong enough to maintain the physical contact and for this they need a frame, which, in the case of neurons, is provided by their skeleton attached to their tiny fingers that will anchor their hands together. My paper describes how molecular fingers that are present in neurons engage the skeleton to react to each other's contact by providing the strength required to hold the contacting neurons' hands and fingers. In my system, the proteins, called latrophilins, constitute the neurons' fingers and come in three different colors, each color finding its match with other fingers from the adjacent neuron that are called teneurins. This color matching allows each neuron containing latrophilins to form unique contacts with neurons containing teneurins and my paper describes how such pairings reshape the hands and arms of cells through their skeleton. Given that the formation of the brain relies on these arm-hand-finger contacts, my paper provides a fresh look at what defines the general framework of this essential organ leading to the establishment of a social network composed of physical contacts between neurons so that they can ‘stick’ together.

**Figure:**
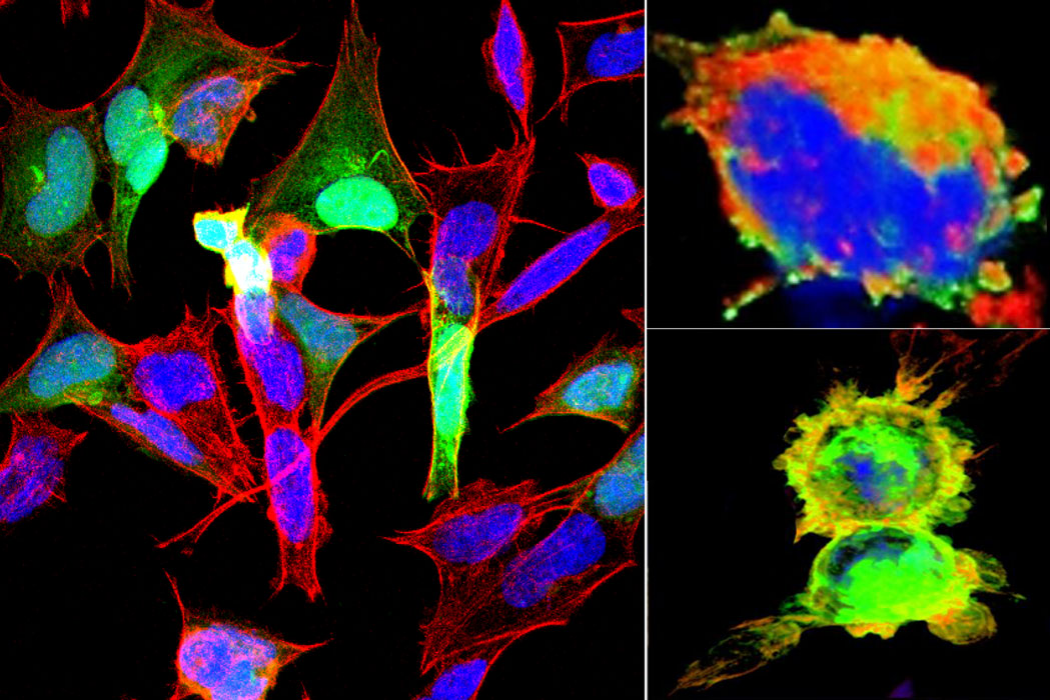
**Latrophilins rearrange the actin cytoskeleton and induce the formation of blebs.**

**What are the potential implications of these results for your field of research?**

Polimorphisms have been found in the genes that encode latrophilins in samples of patients affected by attention deficit and hyperactivity disorder. Understanding the physiological functions of latrophilins allows us to build a conceptual platform on which the pathophysiological roles of these proteins will be tested. The fact that latrophilins are able to regulate the actin cytoskeleton opens a new avenue of research on these receptors and other adhesion GPCRs, since it offers a new focus on their cellular functions. Also, these results potentially rebrand these receptors as frame providers even before their adhesion function comes into play, a paradigm change for this prototypical subfamily of adhesion GPCRs

**What has surprised you the most while conducting your research?**

Life itself… During our experiments we subjected cells to various conditions that could compromise their survival, immediately observing responses; either detectable to the naked eye or registered in some equipment, but always there. It is amazing how fast and precise the cells respond to each stimulus and their great ability to survive and adapt. For example, when we did experiments that involved mechanical stress, the cells immediately formed stress fibers that helped provide additional support; when cells were scrapped to lift them from their plates, their adhesions became stronger; when cells got in touch with each other under conditions of receptor-ligand pairings, it looked like they were looking for one another and sending communication appendices. It is also surprising how all this is regulated by very small molecules which are capable of triggering processes as complex as thoughts, or the functioning of a system or even of a whole living organism.

“It is amazing how fast and precise the cells respond to each stimulus and their great ability to survive and adapt.”

**What, in your opinion, are some of the greatest achievements in your field and how has this influenced your research?**

The discovery of the cytoskeleton as a fundamental organelle for cellular life, through the formation of transport structures, mechanical support, division and cellular communication. Also all the studies on actin and the large amount of associated proteins that allow it to respond quickly to cellular requirements. These previous discoveries guided my intellectual curiosity which resulted in asking the right questions at the right times, as well as providing the relevant methodologies to answer these questions.

**What changes do you think could improve the professional lives of early-career scientists?**

Involve new researchers in scientific events open to the general public and give them the opportunity to present their ideas and develop their projects openly. Contribute constructive criticism to their works and support them to publish them. Open opportunities for exchanges and research trainings in laboratories of different parts of the world. Support them to learn new languages, maybe with open web platforms. Open training courses and scientific training should be constant and accessible to all, for example via web platforms.

“Open training courses and scientific training should be constant and accessible to all.”

**What's next for you?**

I'm halfway through my doctorate. I want to further dissect the signaling pathways involving actin cytoskeleton remodeling through latrophilin activation and transfer the findings to neuronal cultures and animal models. I am determined to understand how this relates to neurodevelopmental defects causing psychiatric disorders and how these findings could help make a dent in pharmacological treatments for example. Of course, there are many steps before reaching this state, but we are optimistic about it. We are also open to new possibilities. When I graduate from my doctorate I am interested in pursuing post-doctoral training and would like to head my own laboratory to continue investigating the molecular basis of intercellular brain communication and how its dysfunction leads to psychiatric illnesses. As a proud Mexican, I would like to transmit my knowledge to up-and-coming young scientists and support them in their formation so that they contribute to society in my country and in other parts of the world. As a mother, wife and currently a scientist-in-training, I have wonderful reasons to continue learning, working and looking for ways to generate scientific knowledge that can be applied in the future and improve the quality of life of people.
